# Impact of Geriatric Consult Evaluations on Hospital Admission Rates for Older Adults

**DOI:** 10.5811/westjem.60664

**Published:** 2023-12-22

**Authors:** Stephen Meldon, Saket Saxena, Ardeshir Hashmi, Amanda Masciarelli McFarland, McKinsey Muir, Fernando Delgado, Isaac Briskin

**Affiliations:** * Cleveland Clinic Emergency Services Institute, Cleveland, Ohio; † Cleveland Clinic Center for Geriatric Medicine, Cleveland, Ohio; ‡ Cleveland Clinic, Department of Quantitative Health Sciences, Cleveland, Ohio

## Abstract

**Introduction:**

We examined the impact of a geriatric consult program in the emergency department (ED) and an ED observation geriatric care unit (GCU) setting on hospital admission rates for older ED patients.

**Methods:**

We performed a retrospective case control study from June 1–August 31, 2019 (pre-program) to September 24, 2019–January 31, 2020 (post-program). Post-program geriatric consults were readily available in the ED and required in the GCU setting. Hospital admission rates (outcome) are reported for patients who received a geriatric consult evaluation (intervention). We analyzed probability of admission using a mixed-effects logistic regression model that included age, gender, recent ED visit, Charlson Comorbidity Index, referral to ED observation, and geriatric consult evaluation as predictor variables.

**Results:**

A total of 9,663 geriatric ED encounters occurred, 4,042 pre-program and 5,621 post-program. Overall, ED admission rates for geriatric patients were similar pre- and post-program (44.8% vs 43.9%, *P* = 0.39). Of 243 geriatric consults, 149 (61.3%) occurred in the GCU. Overall admission rates post-program for patients receiving geriatric intervention were significantly lower compared to pre-program (23.4% vs 44.9%, *P* < 0.001). Post-program GCU hospital admission rates were significantly lower than pre-program ED observation unit admission rates (14/149, 9.4%, vs 111/477, 23.3%, *P* < 0.001). In the logistic regression model, admissions post-program were lower when a geriatric consult evaluation occurred (odds ratio [OR] 0.58, 95% confidence interval [CI] 0.41–0.83). Hospital admissions for older ED observation patients were also significantly decreased when a geriatric consult was obtained (GCU vs pre-program ED observation unit; OR 0.27, 95% CI 0.14–0.50).

**Conclusion:**

Geriatric consult evaluations were associated with significantly lower rates of hospital admission and persisted when controlled for age, gender, comorbidities, and ED observation unit placement. This model may allow healthcare systems to decrease potentially avoidable hospital admission rates in older ED patients.

Population Health Research Capsule What do we already know about this issue?

*Older ED patients have higher admission rates than other age cohorts.*

What was the research question?

*Can an ED-based geriatrician assessment program impact admission rates in older ED and ED observation unit patients?*

What was the major finding of the study?

*Admission rates were lower for patients receiving a comprehensive geriatric assessment (23.4% vs. 44.9%). Geriatric assessment had the largest impact on ED observation patient admissions (9.4% v 23.3%; OR 0.27, 95% CI 0.14–0.50). Controlling for observation status, the odds of admission remained significantly decreased when a geriatric consult evaluation occurred (OR 0.58, 95% CI 0.4–0.83).*

How does this improve population health?

*An ED-based geriatric assessment program may allow healthcare systems to decrease potentially avoidable hospital admission rates in older ED patients.*

## INTRODUCTION

### Background

Older adults have some of the highest rates of emergency department (ED) use in the United States.^1^ ^–^ ^4^ Traditional ED models of care, however, are not ideally suited for the complex clinical presentation and healthcare needs of older adults.^5^ ^–^ ^7^

### Importance

Older ED patients have higher admission rates than other age cohorts.^1^ ^,^ ^4^ Hospitalizations in this group have significant adverse health outcomes including iatrogenic complications, delirium, functional decline, and loss of independence.^8^ ^–^ ^10^ Hospitalizations also result in significant healthcare costs and inconsistent quality of care.^7^ While several studies have evaluated subsequent ED use, subsequent hospitalizations and healthcare utilization following an index ED visit that included a geriatric-trained nurse or advanced practice nurse geriatric assessment,^11^ ^–^ ^14^ few have evaluated the effect of a geriatric assessment program provided by an on-site, ED-imbedded geriatrician on admission rates during an index ED visit.

### Goals of Investigation

Our objective in this study was to measure the impact of a geriatric consult program occurring in the ED setting or within an ED-based observation unit on hospital admission rates in older ED patients.

## METHODS

### Study Design, Setting, and Selection of Participants

This was a retrospective case control study conducted from June 1–August 31, 2019 (pre-program) and September 24, 2019–January 31, 2020 (post-program) that examined the impact of a geriatric consultation program in the ED and ED observation setting on admission rates for older patients. The setting was an academic medical center with approximately 1,400 medical inpatient beds. The ED had 58 acute care beds and approximately 67,000 total annual visits, with 24% geriatric (age ≥65 years) encounters and a longstanding, ED-based observation unit (clinical decision unit [CDU]). The CDU was a short-stay (23 hours), 20-bed observation unit within the ED footprint, designed for additional condition-specific treatment or additional evaluation to determine the need for hospital admission. Clinical staffing of this unit consisted of acute care advanced practice nurses (APN) and unit-dedicated nurses. Emergency department pharmacists were available 24 hours per day, and case management roles were staffed for 14 hours (8 am–10 pm) per day. Well-established CDU guidelines for short-stay ED observation patient selection were used throughout the study period.

A geriatric ED program was implemented with an imbedded geriatric consultant in the ED and ED-based observation unit (post-program) and became the basis for a subsequent geriatric ED accreditation application. As part of the geriatric ED program development, five CDU beds were designated as a geriatric care unit (GCU), although census could vary and was not limited by bed availability. Geriatric consult coverage was provided by the same geriatric physician four days per week and a single geriatric-trained APN one day per week. Geriatric consults were made available in the ED and required for all patients placed in the GCU. The geriatric consult typically included screening for dementia, depression, mobility, assessment of multi-morbidity and social support, and medication review. Case managers in the ED assisted with additional patient service needs such as mobility devices, home physical therapy, and home health services. Geriatric coverage was provided on weekdays from 9 am–5 pm. Patients placed in the GCU off-hours were seen the following day, except on weekends.

As part of the geriatric ED program, emergency physician and advanced practice practitioner (physician assistants and APNs) education was provided that focused on the eight domains of geriatric care competencies model.^15^ In addition, geriatric patient screening and nursing-driven delirium screening were implemented. Although a number of geriatric ED screening instruments exist,^16^ ^,^ ^17^ the performance of such instruments lacks sensitivity and specificity for predicting subsequent healthcare services.^18^ ^,^ ^19^ In addition, it can be difficult to implement these instruments in a high-acuity and high-volume ED setting. For this reason, a modified Delphi approach (which included content experts in geriatrics, geriatric emergency medicine, case management, and pharmacy) was used to select electronic health record (EHR) screening criteria to identify high-risk elders.^20^

Characteristics chosen included age ≥80 years, positive delirium screen, fall presentation or history of falls on triage screening, dementia diagnosis noted in the EHR, polypharmacy (defined as greater than 10 medications), and more than five ED visits within the preceding year. Delirium screening consisted of a two-step process for patients aged 65-79 years. In this group, patients or family members/caregivers were asked whether there was concern for confusion, delirium, or a change in mental status; positive responses resulted in formal screening using the 4AT delirium screen.^21^ In patients ≥80 years, this first-step screen was omitted, and patients in this age group were screened with the 4AT instrument.

To help notify emergency physicians of the geriatric screening results, an EHR-automated best practice alert alerted the physician to high-risk status upon entering the EHR. A positive delirium screen also resulted in an EHR banner informing physicians that the delirium screen was positive. An ED geriatric consult order populated a patient list for geriatric physician and APN use. Consultation and disposition decisions were at the discretion of the ED attending physician and based on their determination of the need for acute medical interventions that clearly required hospitalization, the need for further evaluation in the CDU because of patient complexity or additional investigation regarding safe disposition, or simple discharge. In the GCU/CDU setting, disposition decisions were made by the CDU APNs based on observation evaluation, test results, and consultations. This process was unchanged from prior practice and the pre-program period.

This study was institutional review board exempt.

### Interventions

Geriatric consult evaluations, the intervention, used a standard comprehensive geriatric assessment with attention to medications, fall risk, depression, cognitive status, functional status, and social support, in addition to current and chronic medical issues. Geriatric consult evaluations were available in the ED at the discretion of the attending physician and required for all patients placed in the GCU. Geriatric patients placed in the ED observation unit who did not receive a geriatric consult evaluation were treated as routine CDU patients. We included all completed geriatric consult evaluations occurring in the ED and GCU in the intervention cohort. We used geriatric consult notes for confirmation of a completed consult.

### Measurement and Outcomes

We included all geriatric ED visits for the pre- and post-program periods. Return visits by ED patients were considered a unique ED encounter with a subsequent disposition. Demographics (age, gender), frequency of prior ED visits within the preceding six months, Charlson Comorbidity Index (CCI),^22^ ^,^ ^23^ and ED unit observation placement were recorded.

Hospital admission rates (the outcome) are reported for both pre- and post-program patient cohorts and for patients who had a geriatric consult evaluation (intervention). We developed a logistic regression model to control for patient variables (including placement in the ED observation unit) and evaluate the effect of geriatric consult evaluation on admission rates. Data was abstracted from the EHR (Epic Systems Corporation, Verona, WI).

### Data Analysis

We summarized continuous measures with mean (+/− SD) or median [Q1, Q3] depending on skewness, and categorical variables were summarized with frequency (percentage). We analyzed the differences between pre- and post-intervention encounters with Kruskal Wallis tests for continuous variables and Pearson chi-square test for categorical variables. *P*-values less than 0.05 were considered significant. Odds of admission modeling was analyzed using a mixed-effects logistic regression model. Age at encounter, gender, recent visit, CCI, referral to ED observation, and geriatric evaluation were all included as predictor variables with hospital admission as the outcome. Odds ratios and 95% confidence intervals are reported. Analysis was done in SAS studio v9.4 (SAS Institute, Inc, Cary, NC).

## RESULTS

There were 9,663 unique geriatric ED encounters, with 4,042 occurring pre-program and 5,621 occurring post-program. The overall median age was 73.0 years (68.0, 80.0) and 52.6% were female. Of these patients, 63% were Black and 35% White. Patient demographics and clinical characteristics are shown in Table 1. Emergency Severity Index (ESI) triage levels were similar between cohorts, with the exception of ESI-1. Eighteen (0.45%) patients received a geriatric consult evaluation in the pre-program cohort compared to 243 (4.3%) in the post-program period (*P* < 0.001). Out of 243 post-program interventions, 149 (61.3%) occurred in the GCU.

**Table 1.** Patient demographics and clinical characteristics.

Factor	Total (N = 9,663)	Pre-Program (N = 4,042)	Post-Program (N = 5,621)	*P*-value
Patient age at ED encounter	73.0[68.0,80.0]	73.0[68.0,80.0]	73.0[68.0,80.0]	0.01^b^
ED patient gender				*0.04* ^*c*^
Female	5,079(52.6)	2,075(51.3)	3,004(53.4)	
Male	4,584(47.4)	1,967(48.7)	2,617(46.6)	
ESI triage level^*^				*<0.001* ^*c*^
ESI-1	84(0.87)	20(0.50)	64(1.1)	
ESI-2	1,128(11.7)	456(11.3)	672(12.0)	
ESI-3H	5,993(62.1)	2,496(61.8)	3,497(62.3)	
ESI-3L	1,957(20.3)	843(20.9)	1,114(19.8)	
ESI-4	444(4.6)	209(5.2)	235(4.2)	
ESI-5	46(0.48)	12(0.30)	34(0.61)	
Seen by geriatrician (Intervention)	261(2.7)	18(0.45)	243(4.3)	*<0.001* ^*c*^
ED encounter within prior 6 months?	4,767(49.3)	1,975(48.9)	2,792(49.7)	0.43^c^
Number of ED encounters in the previous 6 months	0.00[0.00,2.0]	0.00[0.00,2.0]	0.00[0.00,2.0]	0.62^b^
ED encounter associated with 10+ medications?	7,067(73.1)	2,966(73.4)	4,101(73.0)	0.65^c^
Charlson Comorbidity Index, mean ±SD	4.23 ± 3.75	4.16 ± 3.65	4.29 ± 3.82	0.09^a^
Medications at time of ED encounter (via Epic)^*^	15.0[10.0,21.0]	15.0[10.0,20.0]	15.0[10.0,21.0]	0.19^b^
Referred to CDU (Observation) within ED?	1,143(11.8)	493(12.2)	650(11.6)	0.34^c^
ED encounter resulted in admission?	4,278(44.3)	1,810(44.8)	2,468(43.9)	0.39^c^
Length of stay (minutes) in ED, not including time in CDU (observation)	304.0[218.0,407.0]	306.0[218.0,408.0]	303.0[218.0,406.0]	0.75^b^

* Data not available for all subjects. Missing values: ESI triage level = 11 (Note: ESI 3H and 3L are internal triage classifications based on use of a fast-track ED model). Medications at time of ED encounter (via Epic) = 121; Statistics presented as mean ± SD, median [P25, P75], N (column %). -values: a = ANOVA, b=Kruskal-Wallis test, c = Pearson chi-square test.

*ED*, emergency department; *ESI*, Emergency Severity Index; *CDU*, clinical decision unit.

Overall, ED geriatric patient admission rates were similar pre- and post-program (44.8% vs 43.9%, respectively; *P* = 0.39). Case mix index was similar in both groups for those patients whose ED encounter resulted in admission (1.3 [0.91, 1.9] pre-program vs 1.2 [0.88, 1.8] post-program, *P* = 0.48). In the post-program cohort, the overall admission rate for both ED and ED observation patients who received a geriatric consult evaluation was lower compared to those patients who did not receive one (23.4% vs 44.9%, *P* < 0.001). We also examined the impact of geriatric consult evaluations on ED observation patients by comparing GCU admission rates with pre-program ED observation unit (CDU) admission rates. Admission rates from the GCU were significantly lower than pre-program admissions from the CDU (14/149, 9.4% v 111/477, 23.3% respectively, *P* < 0.001). As a comparison, admission rates from the CDU in non-geriatric patients was 22.3% pre-program and 23.5% post-program (174/765 vs 226/958, respectively; *P* = 0.74).

The mixed-effects logistic regression model results are shown in Table 2. Odds of hospital admission for ED observation patients were significantly decreased when a geriatric consult evaluation occurred (GCU vs pre-program CDU, OR 0.27, 95% CI 0.14, 0.50, *P* < 0.001). Other predictors associated with admission included age, which had a surprisingly modest but negative effect, male gender, and comorbidity burden (increasing CCI score). Visits to the ED in the prior six months were not a predictor of admission. To assess the impact of the geriatric ED program development and account for possible emergency medicine practice and ED process changes, we examined admissions for the post-program period using the same mixed-effects logistic regression model (Table 3). Controlling for all variables, including ED observation status, the odds of hospital admission were significantly lower when a geriatric consult evaluation occurred (OR 0.58, 95% CI 0.40–0.83, *P* = 0.003). To further assess the effect of the geriatric ED implementation, we also looked at overall admission rates for ED observation patients. Pre-program ED observation (CDU) patients had a 69% higher odds of admission compared to all ED observation (both CDU patients who did not receive a geriatric evaluation and GCU patients) post-program patients (OR 1.686, 95% CI 1.26–2.34, *P* = 0.002).

**Table 2.** Variables impacting hospital admission: logistic mixed-effects model results.

Factor	Estimate	95% Confidence Interval	*P*-value
Age at Encounter	0.979	(0.97,0.988)	*<0.001*
Gender (Male vs Female)	1.333	(1.170,1.518)	*<0.001*
Visit in prior 6 months? (No vs Yes)	0.995	(0.894,1.133)	0.94
CCI	1.461	(1.392,1.533)	*<0.001*
Seen by geriatrician, GCU (vs pre-CDU)	0.266	(0.142,0.500)	*<0.001*
Not referred to ED observation, post (vs pre)	0.756	(0.485,1.178)	0.22

*CCI*, Charlson Comorbidity Index; *GCU*, geriatric care unit; *CDU*, clinical decision unit; *ED*, emergency department.

**Table 3.** Variables impacting hospital admission: logistic mixed-effects model results (post-program patients only).

Factor	Estimate	95% Confidence Interval	*P*-value
Age at encounter	0.979	(0.97, 0.987)	*<0.001*
Gender (male vs female)	1.248	(1.113, 1.400)	*<0.001*
Visit in prior 6 months? (No vs Yes)	1.036	(0.925, 1.161)	0.54
CCI	1.513	(1.449, 1.580)	*<0.001*
Not Referred to ED observation	4.328	(3.413, 5.490)	*<0.001*
Seen by geriatrician (Intervention) (vs not seen by geriatrician)	0.579	(0.405, 0.828)	*0.003*

*CCI*, Charlson Comorbidity Index; *ED*, emergency department.

Summed ED plus observation unit time length of stay (LOS) was higher in the GCU group vs the CDU group by 149 minutes (1,369.0 minutes [117.0–1587.0] vs 1220.0 minutes [936.0–1459.0], *P* < 0.001).

## DISCUSSION

In this retrospective case control study, we demonstrated an associated decrease in hospital admission rates for older ED and ED observation patients who received a geriatric consult evaluation. This effect persisted when we controlled for age, gender, recent ED visits, CCI, and referral to an ED-based observation unit. The overall effect was a 42% reduction in odds of admission. For ED observation patients, the impact of the geriatric evaluation was even more significant with a 73% reduction in the odds of admission. Our results add to previous studies that have examined geriatric interventions and the impact on index ED visit hospital admission. Prior studies have shown a mixed-effect of ED geriatric intervention, with either decreased,^13^ ^,^ ^14^ ^,^ ^24^ unchanged^25^ ^,^ ^26^ or even increased subsequent healthcare utilization.^7^ These discordant results likely reflect patient heterogeneity, availability of follow-up and community resources, and individual emergency physician practice and ED site-specific processes.

Hwang et al. examined the effect of an ED-based transitional care nurse (TCN) on inpatient admission during the index ED visit at three sites that used the Geriatric Emergency Department Innovations in Care through Workforce, Informatics, and Structural Enhancements (GEDI WISE) model of care. The GEDI WISE TCN intervention focused on facilitating transitions of care and avoiding inpatient admissions when possible. Admission reduction varied between 4.7–16.5%.^28^ Another GEDI model reported 13% fewer admission following a GEDI Nurse Liaison assessment. An increased ED length of stay (LOS) and possibility of selection bias (ESI triage scores were used to compare cohorts) were noted by the authors.^29^ Similar findings were noted in a pragmatic trial using the GEDI model. An increased likelihood of discharge (hazard ratio 1.19) and, conversely, a reduced ED LOS following GEDI team evaluation were noted.^30^ A planned subgroup analysis of this study examining ED discharge for patients of residential aged care facilities showed similar results.^31^

A non-randomized prospective study using a geriatric allied health services care coordination team found a much more modest 2.4% absolute reduction in admissions in the intervention group, which was limited to a small number of common presenting problems, such as musculoskeletal conditions.^32^ Keyes et al also examined admission rates following the opening of a geriatric ED and found a modest 3% reduction (47% pre-senior ED and 44% after).^33^ This model included ED staff education and training and a case management approach but did not use geriatricians. Our geriatric ED program differs from these models because of an imbedded geriatric physician and APN. This integrated geriatric consultation intervention was thus available on-site and in real time during the ED and ED observation unit evaluation.

An important consideration, with both our program and others, is careful patient selection, with a focus on targeted evaluation of older patients who do not obviously require hospital admission on initial ED evaluation. The concept of “high complexity, low acuity” is useful to describe this patient population. It seems likely that geriatric screening tools, combined with geriatric assessments and well-developed transitions of care programs, would have the greatest impact on potentially avoidable hospitalization rates in older patients.^34^ ^,^ ^35^ A comprehensive geriatric assessment is designed to evaluate and address functional status, cognitive status, polypharmacy, falls assessment, social support, and other geriatric issues that are difficult to assess in the usual ED setting. Faced with these time-consuming, complex patients, emergency physicians often err on the side of admission.

Addressing these concerns with a comprehensive geriatric assessment and using safe transitions of care can potentially reduce hospital admissions for this high-complexity, vulnerable population. We believe our results are due to this direct geriatric physician/APN assessment, coupled with an existing transitions of care program and appropriate patient selection. This is supported by our data which showed a larger impact of geriatric intervention in our older ED-based observation (GCU) patients.

The advantages of an observation-based geriatric ED model are numerous, including use of existing ED space and staffing, easier use of defined geriatric protocols, decreased impact on ED throughput, and additional professional billing for both emergency physicians and geriatric consultants. ^5^ ^,^ ^36^ Our summed ED-observation unit LOS was higher in the GCU cohort, but the difference of 149 minutes, in our view, had no appreciable impact on operations of the ED observation unit. Neither was there significant ED operational or throughput impact since the observation unit is not used as additional ED clinical space.

Interestingly, our older post-program CDU observation patients who did not receive a geriatric evaluation (non-GCU) also had a lower admit rate; so it appears that our geriatric ED program had a positive overall effect on admission rates. Our institution does have a transitions of care program initially developed for accountable care organization (ACO) patients, which uses ED case management staff.^37^ As this program has evolved, it has become increasingly payer-agnostic and has been applied to a broader number of insured patients. It is likely that as experience with the geriatric ED program developed, this existing transition of care pathway was used for non-ACO geriatric patients. In addition, additional education and experience around geriatric syndromes and domains likely increased the comfort level of APNs and physicians and allowed for discharge home with additional follow-up and services, thereby avoiding potential admissions.

In this analysis, we did not analyze the impact of geriatric intervention on subsequent ED visits or subsequent hospitalization following the index ED visit. Further evaluation is planned to determine whether this geriatric intervention also has an impact on subsequent ED visits or hospitalization and whether this geriatric ED program affected patient experience.

From a financial policy perspective, avoiding potentially avoidable hospitalizations can have important consequences for patients, insurers, and healthcare systems. This is especially relevant for value-based contracts and ACOs. We believe our compelling results can be attributed to our model, which included real-time geriatric consultation. However, we recognize that staffing model costs and cost effectiveness are important considerations when adopting a geriatric model of care.^5^ ^,^ ^38^ As noted, other geriatric ED care models assess mobility and functional status, cognition, depression, and other geriatric syndromes using nursing or case management personnel and standardized screening tools. Future studies should compare the cost effectiveness of different geriatric ED models of care, examine healthcare outcomes and additional healthcare utilization, and measure financial impact from a healthcare system perspective.

## LIMITATIONS

Limitations of this study include the single-site, academic medical center setting and patient selection bias for geriatric consults and GCU placement. Geriatric consults were not available on weekends and off-hours weekdays. The high-risk criteria were automated and EHR-driven and remained consistent during the post-program period; however, we did not perform independent verification of our selected high-risk criteria. In addition, delirium screening was not always completed. Neither did we compare ED clinical impressions or CDU admitting diagnosis for the pre- and post-program cohorts.

To help address selection bias and cohort differences, our logistic regression model controlled for age, gender, patient comorbidities, recent ED visits, and ED observation placement, in addition to geriatric intervention. We did not include other potentially important demographic variables such as race, education, or income level. We did examine admission rates for the similar populations of geriatric ED observation patients (GCU vs pre-program CDU patients) to help quantify the impact of the geriatric intervention on patients who did not initially require hospital admission. The effect of the intervention is also supported by the stable CDU hospital admission rates in non-geriatric CDU patients during the study periods. Even with these efforts, some selection bias in obtaining geriatric consult evaluations or placement in the ED-observation unit is likely present which may limit the magnitude of our results.

Emergency medicine practice or process changes between pre- and post-program also may account for some differences. Although our ED has a previously developed transitions of care program, case management staffing and transitions of care processes were unchanged during the pre- and post-program periods. In addition, pre- and post-programs occurred at different times in the year and could reflect seasonal variability in illness patterns and subsequent admission rates.

Of note, the overall ED geriatric admission rates were similar pre- and post-program. This may reflect the small percentage of patients (4.3%) who received a geriatric consultation post-program intervention. While this is significantly greater than pre-program, it represents an opportunity to increase the scope and scale of the program in the future. Increasing the number of older patients who receive and benefit from the intervention would likely impact overall ED geriatric admission rates. Last, this program was part of a Geriatric ED accreditation application and used hospital resources (program managers, analytics) and philanthropic support, which could limit replication and generalizability.

## CONCLUSION

Implementation of this novel geriatric consultation program in the ED and an ED-based observation unit was associated with significantly decreased odds of hospital admission in high-risk, lower-acuity older patients. Use of an ED or ED observation unit-based geriatric physician or advanced practice nurse consult program may allow healthcare systems to decrease potentially avoidable hospital admissions from the ED in older adults.
